# Supplementary material for: EnzML: multi-label prediction of enzyme classes using InterPro signatures
Source: BMC Bioinformatics. 2012 Apr 25;13:61. doi: 10.1186/1471-2105-13-61 (PMC3483700; doi:10.1186/1471-2105-13-61)
Supplement: Addtional file 5 — The Java code to format the data files, evaluate and predict. The file enzml_java_code.tar.gz contains the Java code used to format database data to ARFF and XML formats, to execute cross and train-test (jackknife) evaluations and to record evaluation results to database. More information is included in the readme.txt file and the Javadoc files. The code can be used with a MySQL database. To use a different database software, other JDBC drivers might be required. [file 1471-2105-13-61-S5.gz › java_code/enzml2011/doc/uk/ac/ed/inf/enzml/weka/IdentifiedSparseInstance.html]

IdentifiedSparseInstance


---


|  |  |  |
| --- | --- | --- |
| **Overview** \| **Package** \| **Class** \| **Use** \| **Tree** \| **Deprecated** \| **Index** \| **Help** | |  |
| **PREV CLASS**   **NEXT CLASS** | **FRAMES**    **NO FRAMES**     **All Classes** |  |
| SUMMARY: NESTED \| FIELD \| CONSTR \| METHOD | DETAIL: FIELD \| CONSTR \| METHOD |  |


---


## uk.ac.ed.inf.enzml.weka Class IdentifiedSparseInstance

```
java.lang.Object
  weka.core.AbstractInstance
      weka.core.SparseInstance
          uk.ac.ed.inf.enzml.weka.IdentifiedSparseInstance
```

**All Implemented Interfaces:**: java.io.Serializable, weka.core.Copyable, weka.core.Instance, weka.core.RevisionHandler

---

``` public class IdentifiedSparseInstance extends weka.core.SparseInstance ```

Class

**Version:**
:   23 Jun 2011

**Author:**
:   Luna De Ferrari luna.deferrari-at-ed.ac.uk

**See Also:**: Serialized Form

---

**Constructor Summary**

`IdentifiedSparseInstance()`


**Method Summary**

**Methods inherited from class weka.core.SparseInstance**

`copy, getRevision, index, locateIndex, main, mergeInstance, numAttributes, numValues, replaceMissingValues, setValue, setValueSparse, toDoubleArray, toStringNoWeight, value`

**Methods inherited from class weka.core.AbstractInstance**

`attribute, attributeSparse, classAttribute, classIndex, classIsMissing, classValue, dataset, deleteAttributeAt, enumerateAttributes, equalHeaders, equalHeadersMsg, hasMissingValue, insertAttributeAt, isMissing, isMissing, isMissingSparse, numClasses, relationalValue, relationalValue, setClassMissing, setClassValue, setClassValue, setDataset, setMissing, setMissing, setValue, setValue, setValue, setWeight, stringValue, stringValue, toString, toString, toString, value, valueSparse, weight`

**Methods inherited from class java.lang.Object**

`equals, getClass, hashCode, notify, notifyAll, wait, wait, wait`

**Constructor Detail**

### IdentifiedSparseInstance

```
public IdentifiedSparseInstance()
```


---


|  |  |  |
| --- | --- | --- |
| **Overview** \| **Package** \| **Class** \| **Use** \| **Tree** \| **Deprecated** \| **Index** \| **Help** | |  |
| **PREV CLASS**   **NEXT CLASS** | **FRAMES**    **NO FRAMES**     **All Classes** |  |
| SUMMARY: NESTED \| FIELD \| CONSTR \| METHOD | DETAIL: FIELD \| CONSTR \| METHOD |  |


---
